# Contraceptive access and use among women with migratory experience living in high-income countries: a scoping review

**DOI:** 10.1186/s12889-024-19778-y

**Published:** 2024-09-20

**Authors:** P. Gozzi, M. Persson, A. Nielsen, H. Kilander, A. E. Kågesten, K. Emtell Iwarsson, D. Ljungcrantz, M. Bredell, E. C. Larsson

**Affiliations:** 1 Department of Global Public Health, Global and Sexual Health, Karolinska Institutet, Stockholm, Sweden; 2 Department of Public Health Sciences, Stockholm University, Stockholm, Sweden; 3 Center for Epidemiology and Community Medicine, Region Stockholm, Sweden; 4 Department of Women’s and Children’s Health, Karolinska Institutet, Stockholm, Sweden; 5 Jönköping Academy for Improvement of Health and Welfare, School of Health and Welfare, Jönköping University, Jönköping, Sweden; 6 Department of Women’s and Children’s Health, Karolinska Institutet, and the WHO Collaborating Centre, Karolinska University Hospital, Stockholm, Sweden; 7 The Public Health Agency of Sweden (Folkhälsomyndigheten), Stockholm, Sweden

**Keywords:** Sexual and reproductive health and rights, Contraception, Family planning, Migration, Healthcare access

## Abstract

**Background:**

Women who have migrated often encounter difficulties in accessing healthcare and experience inequitable sexual and reproductive health outcomes in destination countries. These health inequities include contraceptive access and use. To better understand what influences contraceptive access and use, this scoping review set out to synthesize the evidence on contraceptive access and use and on associated interventions among women with migratory experience in high-income countries (HICs) in Europe, North America and Australasia.

**Methods:**

The scientific databases PubMed, Web of Science and CINAHL were searched for peer-reviewed quantitative, qualitative and mixed method articles published between January 2000 and June 2023. Articles were included if they reported on studies exploring contraceptive use to prevent pregnancies among women of reproductive age with migratory experience living in HICs. Two researchers independently screened and extracted data from the articles. Findings were categorized by patient and health system level factors according to Levesque et al.’s framework of access to health care.

**Results:**

A total of 68 articles were included, about half (*n* = 32) from North America. The articles focused on the individual level rather than the health system level, including aspects such as women’s contraceptive knowledge, the influence of culture and religion on accessing and using contraception, partner involvement, and differing health insurance coverage. On the health system level, the articles highlighted lack of information on contraceptive services, cultural (in)adequacy of services and communication aspects, contraceptives’ side effects, as well as geographic availability and cost of services. The review further identified three articles reporting on interventions related to contraceptive counselling.

**Conclusions:**

There is a lack of knowledge regarding how health systems impose obstacles to contraceptive services for women with migratory experience on an organizational level, as research has focused heavily on the individual level. This review’s findings may serve as a foundation for further research and advances in policy and practice, specifically recommending early provision of health system related information and contraceptive education, engagement of male partners in contraceptive discourses, cultural competency training for healthcare professionals, and strengthening of interpretation services for contraceptive counselling.

**Supplementary Information:**

The online version contains supplementary material available at 10.1186/s12889-024-19778-y.

## Background

In 2018, the Guttmacher-Lancet Commission defined sexual and reproductive health and rights (SRHR) as a *“state of physical, emotional, mental and social well-being in relation to all aspects of sexuality and reproduction”*, where *“all individuals have a right to make decisions governing their bodies and to access services that support that right”* [1]. Migrants and refugees have been described as groups facing particularly precarious conditions concerning SRHR and access to associated services [1–3].

International migration is rising globally due to political and economic events, conflict, and climate change. In 2020, the International Organization for Migration, the United Nations’ migration agency, recorded 281 million international migrants and over 30 million refugees and asylum seekers, of which women made up nearly half [4, 5]. The largest destinations for international migration are Europe and Asia comprising almost two thirds of migrants, while North America counts another 20.9%; among the top 20 destinations of international migrants eleven countries are considered high-income economies [4]. Migrant women are particularly vulnerable to marginalisation in the health sector due to structural and gender inequalities, putting them at risk of negative experiences and adverse health outcomes [2, 3]. Provision of sexual and reproductive health (SRH) care and maternal care to migrant populations has been reported to be less and/or delayed compared to majority populations [6, 7]. Research also shows that migrant and refugee women living in high-income countries (HICs) have higher maternal mortality rates, higher rates of unintended pregnancies and of induced abortion than majority population women [6, 8–11]. Therefore, it is evident migrant and refugee women have distinct needs that warrant in-depth understanding to protect their right to equitable SRHR. This also implies access to non-discriminatory, available, accessible, acceptable and quality contraceptive services, in alignment with the Sustainable Development Goals (SDGs) 3 and 5 of the Agenda 2030 [12, 13].

Contraceptives prevent unintended pregnancies and pregnancy-related health risks, and allow women and girls to keep pursuing education and employment [14, 15]. Previous research reports high unmet contraceptive needs among refugee populations [16], and low rates of contraceptive use among migrant women in HICs [6, 17–19]. In a retrospective study among refugee women in Canada, almost one third did not use contraception and the unmet contraceptive need in that population was higher than reported globally or for Canadian estimates [16]. In Finland, foreign-born women had double the risk for contraceptive non-use prior to induced abortion compared with Finish-born women [19]. Similarly, immigrant women in Sweden born outside of the country reported having less experience of contraceptive use and exhibited higher frequencies of induced abortion compared to women born in Sweden [18].

Previous research has thus far been descriptive and focused on contraceptive prevalence and use of different methods, frequently drawing comparisons to majority populations [16, 18–20]. Prior reviews have shed light on migrant women’s access to healthcare including SRH in specific geographic contexts or among population subgroups [21–25]. Yet, contraceptive access and its determinants have been explored to a lesser extent. Thus, this review aims to synthesize what has been reported on contraceptive access and use, as well as associated interventions among women with migratory experience in HICs in Europe, North America and Australasia. Placing this study in the context of HICs intended to reduce the knowledge gap for countries who, in view of their access to resources, should and can be held accountable for their provision of healthcare. However, it is also recognized that settings missed by this eligibility criterium provide not only refuge to many migrating populations but are also prolific in conducting research relevant to improving their well-being. Furthermore, the term “women with migratory experience” is here understood to include migrants, refugees and asylum seekers.

### Analytic framework

**Fig. 1:**
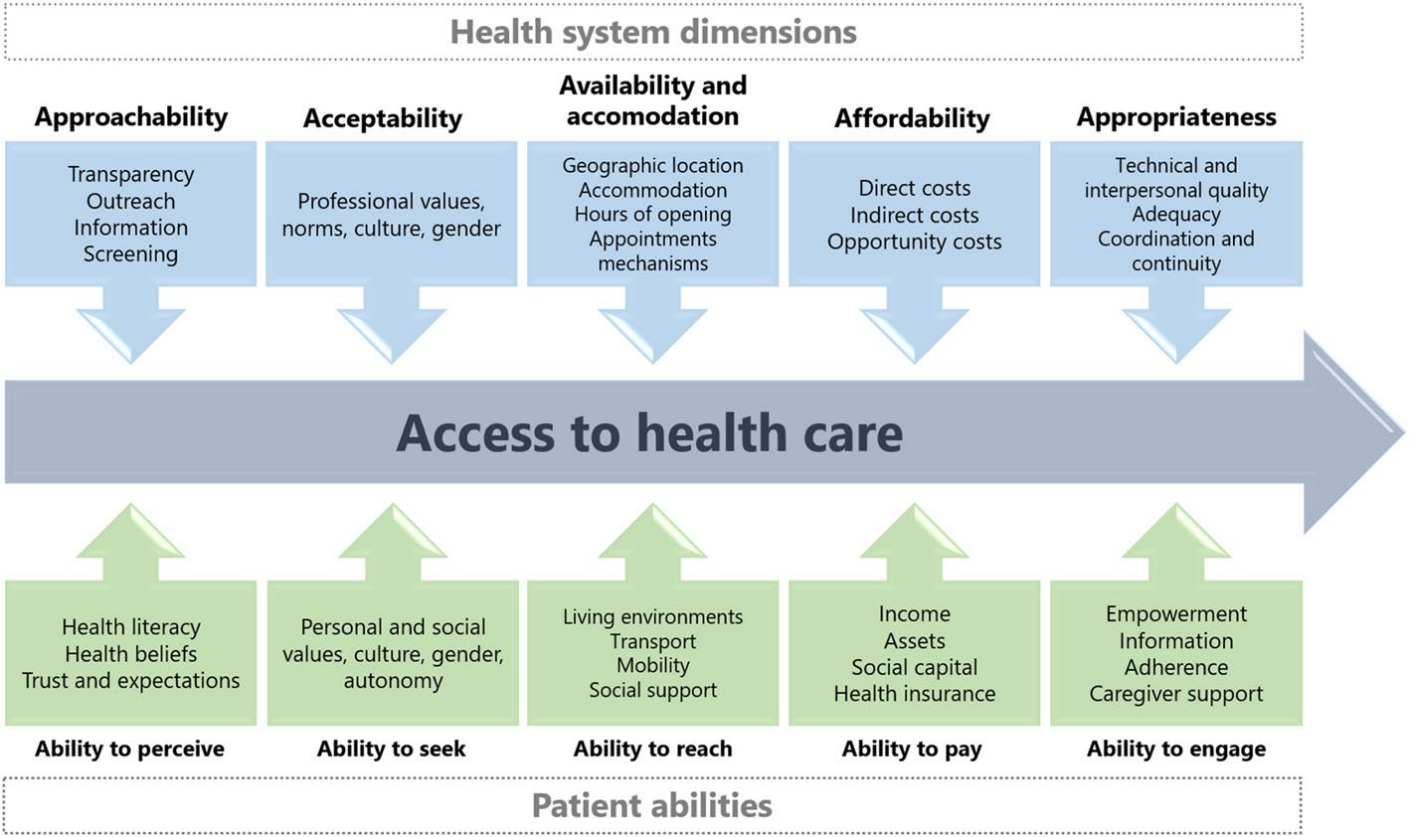
Framework of access to health care, adapted from Levesque et al. [26]

Levesque et al.’s framework on access to health care was used to summarise the findings of this review. Here, access to health care is defined as “*the opportunity to reach and obtain appropriate health care services in situations of perceived need for care*” [26]. The framework dissects access to health services on a health system (supply-side) and patient (demand-side) level (Fig. 1). Determinants on both sides are understood to interact and to shape the progression towards accomplished healthcare access (i.e., utilization). On the organizational health system level, Levesque et al. identify five dimensions of accessibility of services: approachability, acceptability, availability and accommodation, affordability, appropriateness. The patient experience on the corresponding demand-side level of the framework is described by abilities of individuals to access health services: the ability to perceive, seek, reach, pay and engage. Despite these domains of individual abilities, it is important to note that Levesque et al. also acknowledge that demographic, social and economic context shape a woman’s access to care. The framework’s overall integrative approach motivated its use in this review (see Additional File 1 for operational definitions).

## Methods

In this scoping review the framework developed by the Joanna Briggs Institute (JBI), as well as the Preferred Reporting Items for Systematic Reviews and Meta-Analyses – Extension for Scoping Reviews (PRISMA-ScR) guidelines were followed to ensure reporting thoroughness [27, 28]. JBI’s “PCC” mnemonic (population, concept, context) was used a priori to specify the review’s subject and outline eligibility criteria [28]. The mnemonic defines a “population” in terms of specific characteristics of the review’s included study participants; it specifies a phenomenon or outcome of interest as “concept”; and it outlines a particular geographic location, cultural and/or social setting as “context” [28]. The inclusion criteria were refined throughout the review process.

### Population

Articles focusing on women of reproductive age (15–49 years) with migratory experience, understood as first-generation, were included. Populations with specific characteristics and needs, such as sex workers, were not included as it was beyond the scope of this review to discuss the experiences that particular groups might encounter. Additionally, articles involving healthcare professionals (HCPs) engaged in contraceptive counselling and provision to women with migratory experience were also included.

### Concept

Articles were included when reporting on contraception to prevent pregnancy (i.e., excluding articles on the use of contraception to prevent sexually transmitted infections only), discussing influences on their access and use, and on interventions targeting contraceptive access and use. Articles on emergency contraception were not considered.

### Context

Articles from HICs in Europe, North America and Australasia according to the World Bank Group’s classification of countries’ income levels [29] were considered for review. This choice was made supposing that those countries have health systems with availability of healthcare, including contraception, and that they would compare with regard to their parallels in observed (im)migration patterns related to geoeconomic trends [4].

#### Further eligibility criteria

In addition to the abovementioned PCC criteria, articles were selected based on further inclusion and exclusion criteria, which are shown in Table 1.

**Table 1:** Further eligibility criteria

Inclusion criteria	Exclusion criteria
▪ Original, peer-reviewed articles ▪ Quantitative, qualitative, mixed methods articles ▪ Published from January 2000 to June 2023 ▪ English language publications ▪ Availability of full text	▪ Secondary literature, grey literature, editorials, letters, commentaries, book reviews, meta-analyses ▪ Articles from low, lower-middle and upper-middle income countries ▪ Articles not discussing contraception as a method to prevent pregnancy ▪ Articles only providing data on contraceptive use and method prevalence ▪ Articles with unclear description of methodology or population (when lacking disaggregation regarding migration status or gender)

The review protocol was registered on the Open Science Framework on 22.03.2023, doi.org/10.17605/OSF.IO/BNSXG.

#### Search strategy

A literature search was performed in the following databases: Medline, Web of Science and CINAHL. After the original search was performed on 21.02.2023, the search was updated on 30.06.2023, using the methods described by Bramer et al. [30]. The search strategy was developed in Medline (Ovid) in collaboration with librarians at the Karolinska Institutet University Library. For each search concept, Medical Subject Headings (MeSH-terms) and free text terms were identified. The search was then translated, in part using Polyglot Search Translator [31], into the other databases. The strategies were peer-reviewed by another librarian prior to execution. De-duplication was performed using the method described by Bramer et al. [32]. One final step was added to compare DOIs. Reference indices of eligible articles were checked for additional relevant literature with the help of the online open-source tool “citationchaser” [33]. The full search strategies for all databases are available as a supplement (Additional File 2).

#### Article screening and selection

All identified references were imported into the research collaboration platform *Rayyan* [34]. After removing any remaining duplicates, publications were screened for relevance to the study aim first by title, then by abstract, and lastly by their full text. Titles were screened by PG, abstracts and full texts were screened by PG and MP independently, while authors EL and AN were consulted on conflicts and ambiguities.

#### Data charting and synthesis

The included articles were charted by categories of descriptive indicators (first author, year of publication; country; study aim/objective) and methodological indicators (method; study design; data collection method and year; population) using Microsoft Excel. Articles were analysed deductively and findings coded and categorised according to health system dimensions and individual abilities of Levesque et al.’s framework of access to health care (Fig. 1) [26]. For text that could not be coded directly in line with the framework, data were classified into the overarching category of “Sociodemographic factors”. Results were reconciled in a summary of the key findings using a narrative approach. Descriptive information from the articles were organised in Microsoft Excel, including frequencies of categories to ascertain where the research focus has so far been concentrated and what findings are most represented in the literature.

#### Departure from protocol

The original aim “to synthesize the evidence for factors impacting contraceptive access and use, and associated interventions among women with migratory experience in high-income countries in Europe, North America and Australasia” was broadened in the course of data extraction by excluding the term “factors impacting”, as impact on contraceptive access and use was not always declared explicitly in the included articles.

## Results

**Fig. 2:**
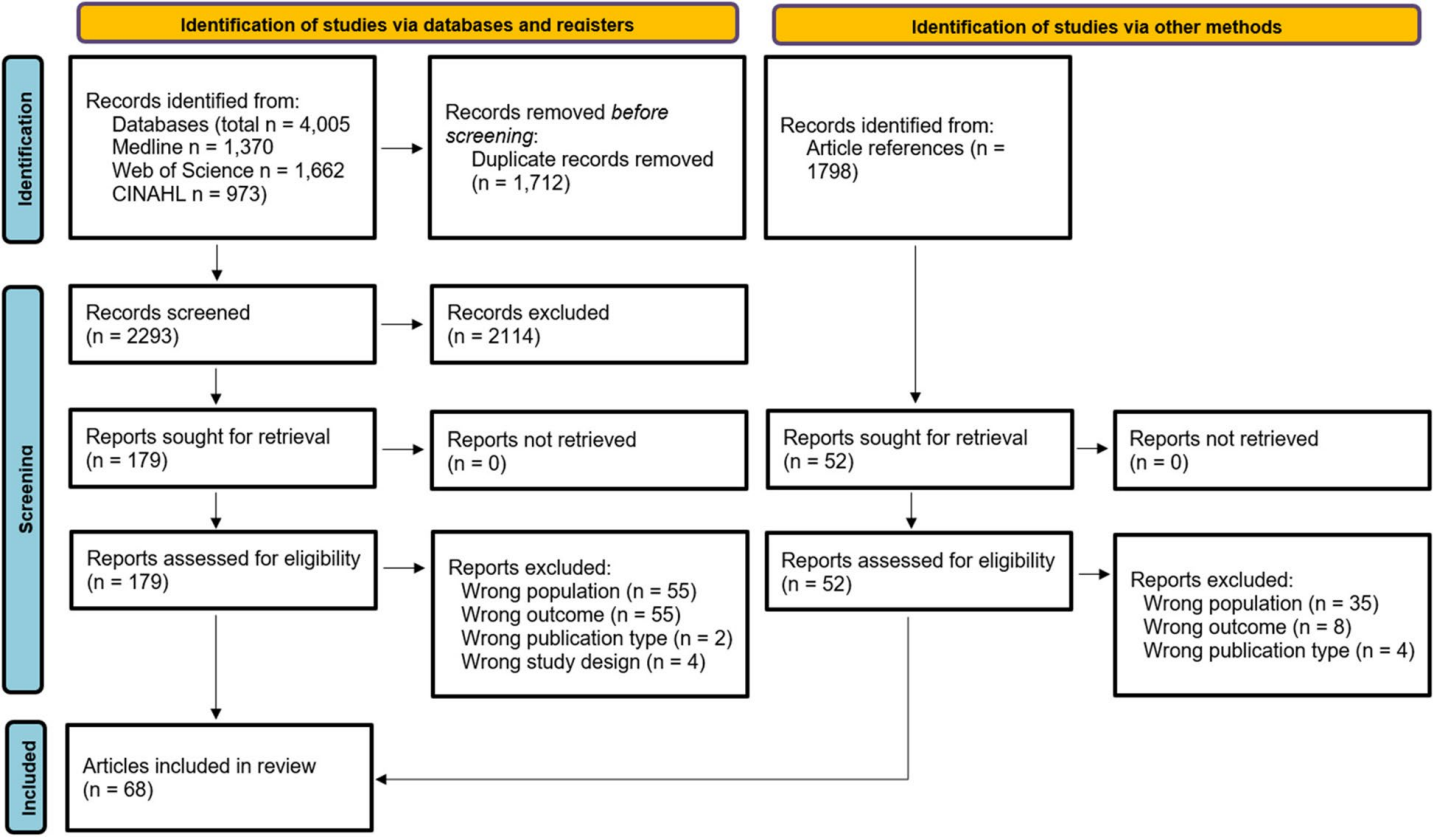
Flowchart of article screening process, based on the updated PRISMA guidelines [35]

From the initial 4005 identified records, duplicates were removed to result in 2293 articles screened by title, and in a second step, 456 articles were screened by abstract. 179 articles were eventually reviewed by full text, of which 63 were eligible for inclusion. An additional five articles were identified by reference searching. In total, 68 articles were included (Fig. 2).

### Characteristics of included articles

**Fig. 3:**
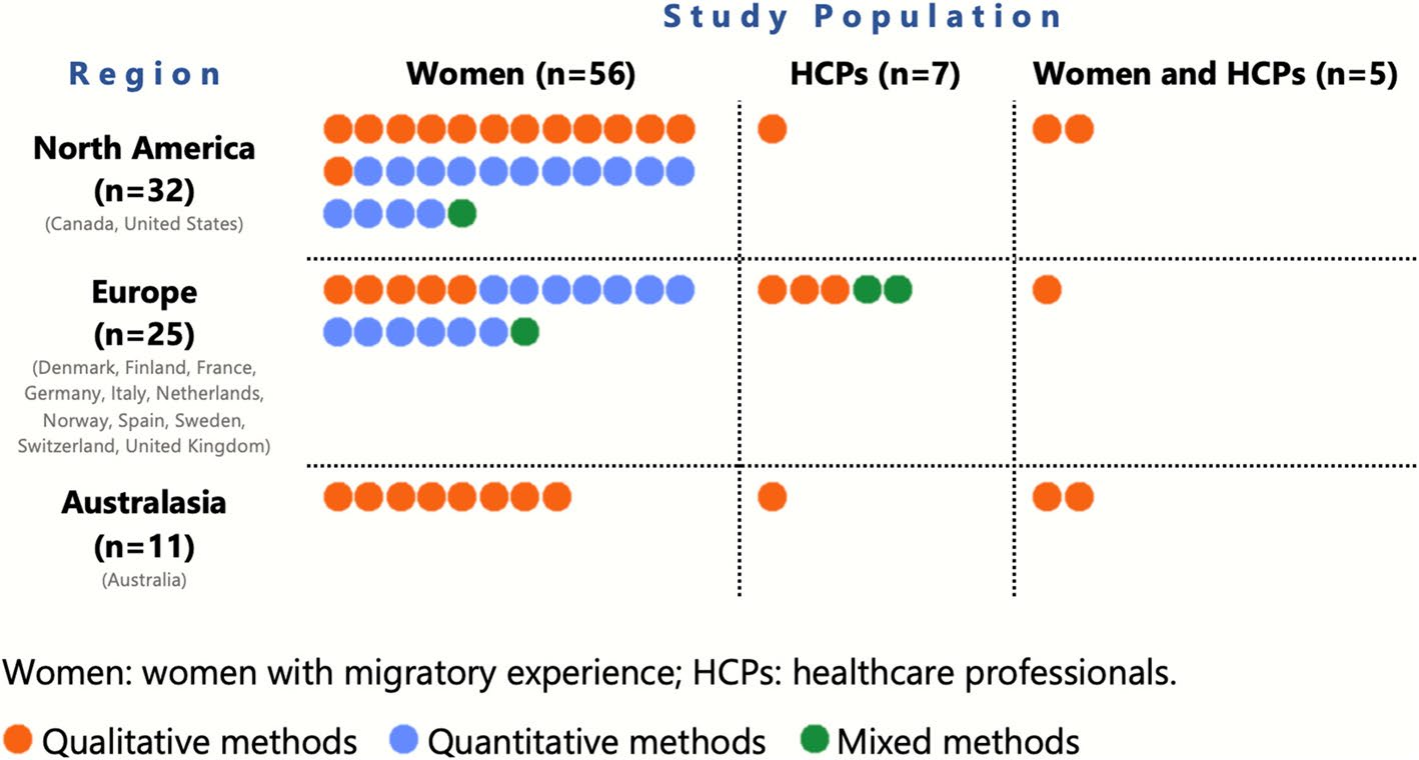
Distribution of articles by region, study population, and method (total *n* = 68). Each disk represents one publication

Almost half (*n* = 32) of the included articles were from North America, of which 28 were based on studies conducted in the United States (U.S.). Twenty-five articles were from Europe (*n* = 25) with Sweden (*n* = 9) being the most common study setting, and 11 articles were from Australasia, which all focused on Australia (Fig. 3). The year with the highest number of publications was 2020 (*n* = 9). Qualitative methods were employed in 36 articles, 28 used quantitative methods, and four used mixed methods (Fig. 3). Only three eligible publications described an intervention.

The majority of the articles focused on women with migratory experience (*n* = 56), seven articles focused on HCPs and five articles included both women and HCPs (Fig. 3). The populations were defined as “immigrant” (*n* = 20), “refugee” (*n* = 12), “migrant” (*n* = 10) or “foreign-born” (*n* = 8). Articles described their population in terms of nationality; the most frequently studied groups were women born in Somalia (*n* = 8) and Mexico (*n* = 6). Some articles characterised their population as “Hispanic” (*n* = 5) or “Latina” (*n* = 4), though this referenced ethnic minorities. The most common types of HCPs were “midwives” (*n* = 5) or described more generally as “healthcare providers” (*n* = 4).

### Findings related to Levesque et al.’s framework

**Fig. 4:**
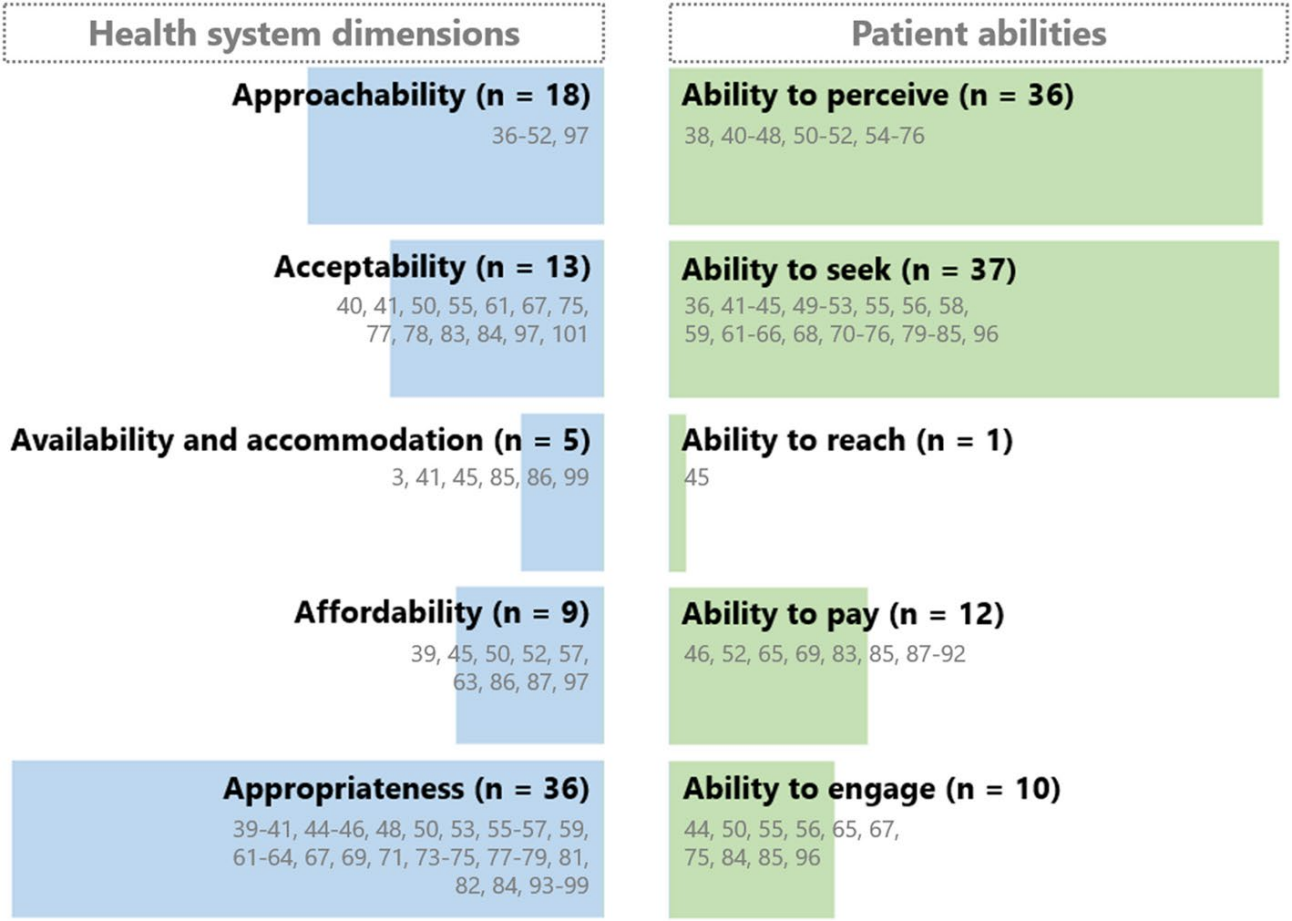
Number of articles referring to dimensions and abilities of Levesque et al.’s framework (with respective references). One article can report results on multiple dimensions and abilities

A majority of the articles investigated demand-side contraceptive access and use, i.e. women's abilities to access care according to Levesque et al.’s framework (Fig. 4). Women’s “ability to perceive” the need for care (*n* = 36) and the “ability to seek” care (*n* = 37) related to contraception were mentioned repeatedly. Articles reporting on health system level factors mainly focused on the “approachability” (*n* = 18), “acceptability” (*n* = 13), and “appropriateness” of provided services (*n* = 36). Several articles reported results from both demand and supply side perspectives and included multiple findings relating to several dimensions and abilities as further presented below.

Below, results are presented in relation to health system and patient levels in the order proposed in Levesque et al.’s framework (main results are compiled in Table 2). Thereafter, interventions related to contraception and the additional category of “Sociodemographic factors” influencing contraceptive access and use are presented.

**Table 2:**
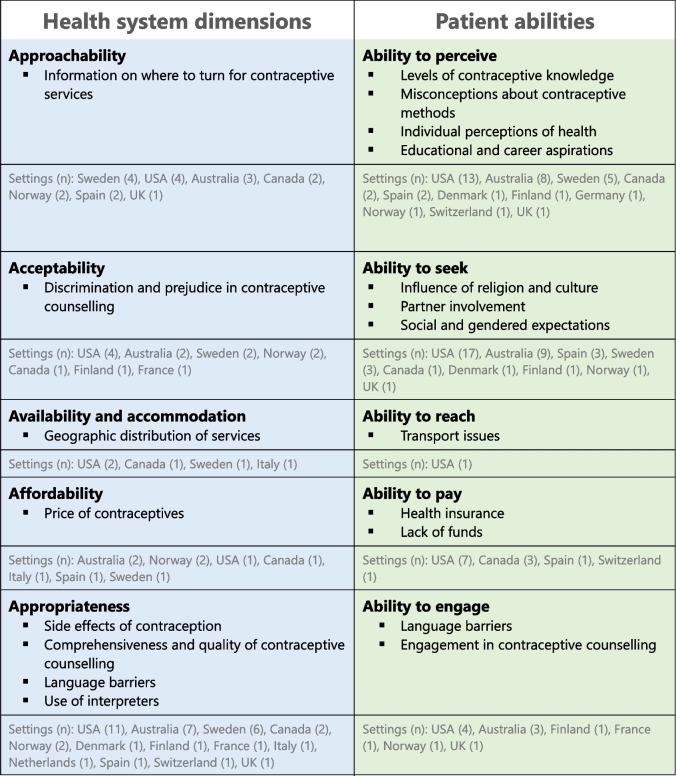
Main findings by health system and patient levels according to Levesque et al.’s framework of access to health care

The number of articles from each country reporting on the respective dimension or ability is shown in brackets (*n*)

#### “Approachability” and “Ability to perceive”: information availability, knowledge and perceived need for care

Sixteen articles described women’s lack of or limited information about where to turn for contraceptive services as a main access barrier [36–51]. Two articles discuss this as related to women’s difficulties in navigating a new health system in the destination country [44, 47]. One cross-sectional Swedish study observed that among Thai-born women, more than a third were not aware of where to receive contraceptive advice [38]. Only one article from Spain reported that immigrant women generally had knowledge on where to procure contraceptives [52].

Limited knowledge on different contraceptive methods, side effects and mechanisms of action among women were presented in 30 articles [40, 41, 43–48, 50, 51, 53–72]. Four articles comparing women with migratory experience to majority-population women found that knowledge levels were lower among the former [54, 58, 64, 66]. Thirteen articles reported on women’s misconceptions about birth control and its side effects, e.g. infertility, cancer, complications during a potential future pregnancy and congenital disorders [44, 45, 47, 50, 51, 55, 61, 63, 66, 68, 70, 71, 73]. Furthermore, six articles found that women often perceived (regular) menstruation as a sign of good health and regarded it as “cleansing” for the body, reinforcing their avoidance of contraceptives that interfere with bleeding patterns [40, 44, 56, 59, 61, 74].

In contrast, five articles observed some or relatively high contraceptive knowledge among women with migratory experience [38, 43, 52, 67, 73]. Further, women gained contraceptive knowledge on their migration journey, such as in refugee camps [43, 67], and/or after settling in the country of destination [44, 49, 51, 63].

Four articles described women’s educational and career aspirations for themselves or their children as motivators to use contraception and drivers in their contraceptive agency [45, 71, 75, 76]. In addition, Soin et al. described how refugee women in the U.S. viewed contraception as a way to regain control over their financial and social instability during displacement [67].

#### “Acceptability” and “Ability to seek”: contextual acceptance of contraceptive services and influences to pursuing care

Both women with migratory experience and HCPs highlighted the importance of HCPs acknowledging and understanding how cultural and religious diversity influence contraceptive decision-making [40, 41, 55, 61, 67]. Out of 11 articles that included the influence of religion and culture on contraceptive use [36, 41, 43, 44, 50, 53, 55, 59, 62, 71, 73], six described religion and culture as barriers to using contraception [36, 43, 53, 62, 71, 73]. One article discussed how religion could either reject contraceptive use or support it, the latter enabling women to adequately care for their children [50]. Articles found that when HCPs framed contraceptive use in terms of birth spacing, contraceptive use would be seen as supported by religious Muslim teachings, though delaying a first birth or limiting the number of children was not acceptable [43, 59, 71, 73]. Furthermore, women with migratory experience encountered discrimination and prejudice in contraceptive counselling [55, 67, 75]. Articles from Finland, Sweden and the U.S. described women being confronted with negative attitudes from HCPs towards their desired number of children [55, 61, 77]; this led to some Somali refugee women using contraceptives (“forcedly”) to avoid further contact with the health system [55]. One article from France pointed at anti-immigrant sentiments against Malian migrant populations, and another article quoted young Latinas in the U.S. and their experiences of meeting “racists” in contraceptive counselling settings [75, 78].

Articles described how considerable involvement of women’s family and friends [51, 62, 63], as well societal expectations interfered with contraceptive decision-making [50, 55, 59, 79]. Ebrahim et al. investigated condom use among Somali and Ethiopian immigrants in the U.S. and found that behaviours of individuals’ social networks were the most significant predictors to condom use [80]. In addition, changes in social circumstances in the country of destination (e.g. lack of childcare support from extended family or women joining the workforce) also contributed to seeking contraceptive care [71, 81]. Spousal involvement and male partner influence in contraceptive decision-making was discussed by 25 articles [41–43, 49–52, 55, 56, 58, 59, 61–66, 68, 72–74, 79, 82–84], of which 18 found partners to be unsupportive of or opposing women’s use of contraception. Women used contraceptives secretly or involved relatives to persuade husbands to approve of contraception [52, 73]. In four articles, women’s decisions regarding contraception were reported not to be influenced by a partner’s position [49, 61, 76, 79]. One qualitative study with immigrant women in Sweden noted that they appreciated their husbands’ presence at contraceptive counselling sessions, and that husbands were perceived as a knowledgeable companions or legal guardians (as they would ensure women had sufficient knowledge prior to making a decision) [61].

Six articles found gendered expectations regarding contraceptive responsibility; women were perceived to carry this responsibility, as reported by women themselves [45, 52, 66, 84, 85] or HCPs [56].

#### “Availability and accommodation” and “Ability to reach”: reachability of services and means to access them

Four articles reported on findings related to this domain [41, 45, 85, 86]. On a health system level, three articles stated that travelling long distances to access services was a barrier to contraception [41, 45, 85], and two articles reported on contraceptive methods not always being available at health centres [45, 86]. Newbold et al. described a situation in which clinics offering interpreters had longer waiting lists, and culturally sensitive clinics were less likely to be close to neighbourhoods with immigrant communities [41]. From an individual’s perspective, one study reported on transport barriers to reach a clinic, such as women not being able to drive, not owning a means of transportation or not travelling out of fear of being stopped and deported [45].

#### “Affordability” and “Ability to pay”: price of contraceptives and insurance coverage

Seven articles showed that the price of contraceptives affected women’s choice of contraception [39, 45, 50, 52, 63, 86, 87]. Alvarez-Nieto et al. observed that among female immigrants in Spain, the perceived accessibility and utilisation of contraceptives were impacted by low or non-existent cost [52]. Other publications reported high costs as deterrent to contraceptive use [39, 45, 50, 63, 87].

Six publications from the U.S. reported results focusing on health insurance [65, 85, 88–91]. Rodriguez et al. compared Medicaid claims, showing that Emergency Medicaid recipients (lower-income noncitizens, i.e. here used as proxy for immigration status) were significantly less likely to receive postpartum contraception compared with Traditional Medicaid recipients, while a policy change in 2018 extending postpartum family planning coverage to Emergency Medicaid recipients led to an increase in their postpartum visits and use of contraception, including different methods [90, 91]. Gurnah et al. described the case of a woman using contraception out of apprehension of losing state support if she had more than four children [83]. One article reported on the Canadian context in which three-month wait periods for a residency-dependent healthcare coverage led to unmet contraceptive need among im/migrant women resulting in unintended pregnancies [92].

#### “Appropriateness” and “Ability to engage”: adequacy of contraceptive services and communication

Fear of side effects, resulting from general knowledge or related to women’s own or their peers’ experiences, was repeatedly named a deterrent from contraceptive use [39, 40, 44, 45, 48, 50, 53, 55–57, 59, 61, 63, 64, 69, 71, 73, 74, 79, 81, 82, 84]. Side effects of hormonal contraception were prominently mentioned, including weight gain and menstrual changes [40, 44, 45, 56, 63, 71]. Articles also reported on preferences for non-hormonal methods and methods that would not interfere with women’s sexual life by inducing irregular or heavy bleeding as a side effect [56, 57, 61, 73, 74].

The extent to which women with migratory experience were provided with counselling and the quality of such counselling were subjects in nine publications [45, 57, 63, 67, 77, 78, 81, 93, 94]. A study from Italy found that when receiving family planning counselling, immigrant women were significantly more likely to use an effective contraceptive method [95]. While two articles found women with migratory experience to be less likely to be counselled on contraceptives as compared to non-migrant women [57, 94], Coleman-Minahan et al. showed that Hispanic foreign-born women were less likely to receive high-quality counselling and more likely to receive no counselling than Hispanic U.S.-born women [93], and White et al. reported on Latina immigrant women in the U.S. not being counselled comprehensively on all methods [45]. Articles detailed experiences of women being prescribed contraception without wanting any, not receiving sufficient information or their wishes being disrespected [63, 67, 78, 81]. One publication described the case of an immigrant woman in the U.S. receiving an unwanted hysterectomy due to the HCP’s failure to inform and educate her on other reproductive options to avoid future pregnancies [77].

Language barriers impairing contraceptive counselling was a common topic, as covered in 12 publications [40, 41, 44, 45, 50, 55, 56, 62, 67, 78, 85, 96]. Interpreters’ role was described as a potential solution and an enabling factor to contraceptive counselling [40], their presence however could also be perceived as embarrassing, discomforting or judgmental by women [41, 61, 67, 84]. Women described feeling apprehensive of attending services due to their lack of language knowledge [85] or not being aware of their right to an interpreter [50]. Two articles reporting from a HCP perspective mentioned concerns about accuracy and completeness of interpreters’ translation [40, 56]. Articles also reported on women experiencing embarrassment when discussing contraception with their HCPs or in front of interpreters [65, 67, 71, 84, 96].

### Interventions

Three articles presented interventional strategies within the scope of contraceptive counselling and provision, of which two were from Sweden and one from Australia (Additional File 4) [97–99]. These studies related to all five health system dimensions (every article mentioned the “Appropriateness” of services) and focused on improvements to contraceptive counselling in terms of service delivery (e.g., scheduling practices, communication, complementary counselling and decision support tools) and identified areas of further potential improvement (e.g., how to better target the dissemination of contraceptive information).

### Sociodemographic factors influencing contraceptive access and use

Overall, 18 articles discussed “Sociodemographic factors” and how they influenced contraceptive access, use and attitude [36–38, 46, 49, 51, 53, 58, 60, 61, 65, 73, 89, 95, 100–103]. Seven articles discussed socioeconomic position and its implications for contraceptive knowledge and choice [38, 49, 53, 73, 89, 101, 102]. Low education levels were associated with a lack of contraceptive knowledge [38] and a reduced likelihood of contraceptive use [49, 102]. Omland et al. identified higher education and being employed as predictors to hormonal contraceptive use for immigrant women in Norway [101]. Findings on (household) income’s influence diverged, from not creating a significant difference regarding contraceptive behaviour to lower income resulting in lower contraceptive use [53, 102]. In the U.S., higher household income and educational status were found to be predictors to favourable contraceptive attitude for Black, foreign-born women [103].

Length of stay in the country of destination was discussed in five articles; longer stay was connected to a higher proportion of women with migratory experience receiving contraceptive counselling. This was a predictor to contraceptive use and increased the likelihood of using a more effective method [36, 46, 60, 101]. Of note, one article from the U.S. did not find length of stay to result in a significant difference in family planning behaviour among African refugee women [53].

Findings on the effect of marital status and parity varied. Four articles found that being married and having children was associated with contraceptive use or utilisation of contraceptive counselling [36, 49, 58, 103]. In addition, pregnancy was described as an entry circumstance to contraceptive counselling among immigrant women in Sweden [61]. Similarly, two articles reported that women received contraceptive information only after a first pregnancy or childbirth [51, 65], and Coleman-Minahan et al. found that parity increased foreign-born women’s likelihood of using effective contraceptives in the U.S. [100]. However, marital status and having children were not significant predictors to utilisation of contraceptive services for immigrant Thai women in Sweden [37], and being married was associated with a less favourable contraceptive attitude among Black, foreign-born women in the U.S. [103]. Further, Lauria et al. did not find a statistically significant association between parity, marital status, education or employment and using effective contraceptive methods among immigrant women in Italy [95].

## Discussion

### Summary of key findings

This scoping review synthesized the current literature on contraceptive access and use among women with migratory experience and on associated interventions in HICs and included 68 articles.

Contraceptive access and use were found to be more frequently studied on the individual level as opposed to the health system level. The majority of the articles focused on women’s ability to seek and reach contraceptive services. In addition, articles mainly reported on barriers, rather than facilitators, to contraceptive access, and only three interventions to improve contraceptive use among women with migratory experiences were identified. The lack of understanding regarding facilitating factors or circumstances was also noted in prior research [104].

The prevailing focus on individual drivers of contraceptive access and use highlights an urgent need to shift research focus towards structural inequalities both within the health system and societies. Limiting research efforts into how health systems operate, could lead to underreporting regarding their responsibilities and potential flaws, thereby unfairly burdening women with undue responsibility. By studying enabling aspects, a better understanding of the health system’s responsibility and the role it plays in women’s contraceptive trajectories can be gained. Furthermore, it might be important to use a participatory approach in which women with migratory experience themselves are involved in all stages of research through co-design, -production and -creation to devise inclusive initiatives [105].

### Information and knowledge – personal health literacy through organisational health literacy

The review identified women’s limited knowledge and misconceptions about contraceptive methods and fear of side effects, particularly of hormonal contraception, as barriers to contraceptive access and use, which is consistent with previous research [21–24, 104, 106, 107]. It is important to mention that the latter concern is not an idiosyncrasy of this population, as a recent systematic review on rejection of hormonal contraception in Western countries recounts [108]. Lack of information on where to access contraceptive counselling services and difficulties in navigating a new healthcare system were also identified frequently, again reflecting prior research [22, 25, 104].

Knowledge on contraceptive methods is crucial for making informed choices, and a more thorough and timely education on contraception for women with migratory experience should be advocated. While this review found that research currently emphasises women’s abilities to access the health system, informed decisions can only be made within an accessible and transparent system. Therefore, health systems’ organizational health literacy, i.e. organisations’ responsibility of making health information accessible and understandable for individuals with differing health literacy skills [109], and its employment should be investigated more thoroughly to allow for its improvement. Health-literate health organisations have been characterised as furthering patient engagement and improving access to care [110]. In previous research, refugees in Sweden communicated a desire for concrete instructions and explanations to empower them in making informed and responsible health-related decisions; they also emphasised the importance of receiving contextual information on the health system to enhance their ability to navigate it effectively [111]. This review also identified pregnancy as an initiator of contraceptive counselling for women with migratory experience, a finding which underscores the delay with which contraceptive services are provided. This indicates a need for early information/education on the health system, ideally provided in early resettlement (e.g., within the scope of civic orientation), and reinforces the need to improve health systems’ organizational health literacy. Accommodating women’s levels of knowledge should be done proactively, not reducing access solely to a result of pre-existing individual health literacy. Outreach activities could further play an important role in ensuring that marginalized groups have access to information about their rights and available services. Establishing access to services will serve as prerequisite for promoting more in-depth SRHR and contraceptive education.

### Partner involvement – from reproductive coercion to gender-transformative family planning practices

A majority of articles referencing male partner or husband involvement in women’s contraceptive journeys reported on opposition or disapproval. This is in line with prior publications, where husbands did not permit contraception and played the dominant role in contraceptive decision-making [22–24]. Such practices can be collocated under the broader concept of reproductive coercion (RC) [112, 113]. RC is considered a form of interpersonal violence acting in either a pregnancy promoting or pregnancy preventing manner, and perpetrators have been identified to be past or current intimate partners, but also family members. While this review identified control over contraceptive choices as a form of RC among women with migratory experience, a literature review previously found immigrant women to be less vulnerable to RC, with the limitation of scant findings [112]. Additional research is required to evaluate impacts of RC on vulnerable populations; resistance strategies such as nondetectable contraceptive methods have been suggested as practical approaches [112].

Chalmiers et al.’s review on refugee women’s experience with contraceptive care discussed that HCPs failed to recognise that some women welcomed partners’ collaboration and support in contraceptive decision-making (which this review found reflected once) [22]. They reported on the transformative manner in which resettlement had led some husbands to being more in favour of joint decision-making [22]. Another qualitative study documented a lack of knowledge on and fear of modern contraceptives among Somali men living in Sweden, while they were cognisant of the usefulness of giving men access to contraceptive education [114]. Furthermore, this review found that contraceptive responsibility was mostly lain on women, when related decision-making was often dictated by men. Including men in contraceptive discourses and educational efforts is understood to promote communication among couples and to be more effective in improving women’s contraceptive use and continuation [115]. Family planning approaches labelled gender-transformative, in that they address and transform gender norms while promoting equity between men and women [116], merit being recognised as opportunity to acquire supportive allies and assets in contraceptive counselling to women and HCPs alike.

### Contraceptive counselling as transcultural interaction

Contraceptive counselling was found to be heavily influenced by discrepancies in language proficiency, cultural and religious aspects and discriminatory attitudes on HCPs’ side. Prior research has commented on miscommunication leading to the undermining of women’s trust in HCPs, instigating contraceptive discontinuation [22–24]. Interpreters are presented as one solution to language barriers; though it is essential to ensure their availability, specifically of female interpreters, appropriate health training, and translation accuracy and reliability. Mere language and technical competence however do not always lead to appropriate communication [117]. Culture-concordance of interpreters who can mediate based on a shared cultural, religious, ethnic background and a common vernacular has been deemed crucial by both migrant populations and HCPs to provide comprehensive care [117, 118], and should be promoted accordingly.

While cultural and religious influence on contraceptive use can vary [22, 23], awareness for their potential relevance should also be heightened. Exemplary, framing family planning practices as “birth spacing” was here found to be acceptable within women’s religious context, and this is supported by two publications reporting on Muslim religious leaders’ endorsement of contraceptive practices to space births for the benefit of the mother’s and the child’s health [119, 120]. Culturally informed and sensitive dialogue in contraceptive counselling settings is crucial to avoid a depreciation of patient-provider interactions that could potentially result in women’s avoidance of seeking contraceptive care. The interventions identified by this review approached contraceptive counselling settings and their service provision by employing communication techniques and additional materials for a better delivery of contraceptive education. The limited number of results clearly indicates a gap in the research and none of the articles reported on interventions associated with significant outcomes in reduced unintended pregnancies or improved contraceptive use.

Discrimination, disrespectful attitudes towards women with migratory experience, as well as women’s stigmatization due to their wish for larger families and HCPs’ tendency to homogenise racialised women were findings of this review, consistent with other authors’ results [22, 23]. Racial discrimination in family planning settings has been documented, where racialised women experience overbearing attitudes and coercion from HCPs in relation to their contraceptive decisions [121, 122]. This review echoed such findings, highlighted by how women's contraceptive needs and preferences were disregarded. Women with migratory experience are affected by marginalising institutional practices and personally mediated forms of racism such as HCP bias, impacting their reproductive health and contraceptive access. While RC was above argued in relation to male partners, HCPs also occupy positions of power regarding women’s reproductive trajectories, and need to be made aware of how that power can be abused. Interestingly, only one article of this review referenced “anti-immigrant sentiments” and merely one relayed women’s wording on “racist” encounters; overall, no article linked instances of discrimination to racism or followed-up on explicit quotes in the discussion. Avoidance to overtly discuss racism in healthcare despite evidence of its existence, increases the risk of perpetuating such issues. With HCPs still perceiving themselves as objective and neutral, and a lack of dialogue on racism in the workplace, the question has been raised how anti-racism training can be implemented successfully while a conceptualisation of how racialisation processes operate in healthcare is not included in such initiatives [123]. Efforts on an organisational level, such as mandatory cultural competency and anti-racism training for HCPs with a clear conceptual and operational rationale, are needed.

### Adopting an intersectional approach

Previous research has addressed health inequities among migrants and explored their social determination, highlighting how health outcomes are subject to gender, occupational status and migrant generation [124]. This review observed results related to education level, (household) income, length of stay in the country of destination and marital status as influences to contraceptive access and use. In addition, the significance of gender roles, as well as racism and discrimination have been discussed. On a public health level, it has been established that analysing singular, individual-level categories, such as class and gender, cannot sufficiently explain health inequalities [125]. Rather the application of an intersectionality lens is appropriate, emphasising the fact that individuals have multiple social identities which interact with one another and with established power systems on a societal level (e.g., racism, sexism). Nevertheless, only one article was informed by an intersectionality approach and 10 articles referenced intersectionality or its applicability in the introduction or discussion. This highlights the need for further examination of intersections of health determinants, as well as the interaction with power systems and their impact on contraceptive access and use. Analysing critical differences within populations could therefore be recommended for future systematic reviews to evaluate which specific population groups are most affected by which determinants to contraceptive access and use, to allow for more specific targeting of future interventions and practices.

### Strengths and limitations

To the best of the authors’ knowledge, this is the first review studying specifically contraceptive access and use among a population of women with migratory experience and on associated interventions in a set of comparable high-income settings. Two factors which strengthened the reliability of this study and thoroughness regarding identification of eligible articles included having the support of a research librarian in developing the search string and two researchers screening the articles independently.

Using an analytic framework founded on pre-existing theory comes with the potential for bias in how information is approached, limiting the identification and interpretation of findings due to preconceived expectations. Overall, Levesque et al.’s framework was perceived by the authors as a comprehensive, fitting template, while it still conceded the identification of an additional category of findings (i.e., “Sociodemographic factors”). Nevertheless, ascribing certain factors to one specific category, or ascribing them to one side of the framework exclusively posed a challenge.

The high number of included qualitative studies is considered a strength as it allows for a thorough examination of experiences and context of women’s contraceptive access. The population of first-generation women was chosen to comprehensively study all women with a personal experience of migration. It should be highlighted that this composite population is, in reality, very heterogeneous and represents many individual experiences and identities among diverse ethnic, cultural, and religious groups. The intent here was to demonstrate the interaction and influence of such variables on contraceptive decision-making and to emphasise their relevance.

Furthermore, articles from the U.S. outnumber publications from other countries, which may bias the information towards conditions experienced in the U.S., particularly regarding aspects that were examined almost exclusively in the U.S. (e.g., health insurance). This could potentially limit the applicability to European or Australian settings.

## Conclusion

This review found that the main reasons for not accessing contraception among women with migratory experience in HICs reported in the included articles were low availability of information on contraceptive services and lack of contraceptive knowledge, male partner involvement and deficiencies in contraceptive counselling. While the research currently focuses on individual-level drivers, there is need for further investigation into health systems’ responsibility and organisational health literacy. Thereby, an increasingly diverse patient population can be equitably provided with health system related information and reproductive education. Further practical advances should target HCPs’ cultural competency, the use of culture-concordant interpreters, and the engagement of male partners in contraceptive discourses. There is a need for more intervention research that measures applicable and significant outcomes. Meaningful interventions might be developed through co-design strategies involving women with migratory experience, HCPs and policy makers to develop comprehensive contraceptive services.

## Supplementary Information

Additional File 1. Operational definitions of framework used in the review.

Additional File 2. Search strategies for all databases.

Additional File 3. Overview of included articles.

Additional File 4. Brief description of identified interventions related to contraceptive counselling.

## Data Availability

Datasets relating to this review are available from the corresponding author upon reasonable request.

## Abbreviations

HCP: Healthcare professional
HIC: High-income country
JBI: Joanna Briggs Institute
RC: Reproductive Coercion
SDGs: Sustainable Development Goals
SRH: Sexual and Reproductive Health
SRHR: Sexual and Reproductive Health and Rights

## Data availability

No datasets were generated or analysed during the current study.
