# Supplementary material for: Contraceptive access and use among women with migratory experience living in high-income countries: a scoping review
Source: BMC Public Health. 2024 Sep 20;24:2569. doi: 10.1186/s12889-024-19778-y (PMC11414253; doi:10.1186/s12889-024-19778-y)
Supplement: Supplementary file 1 — Additional File 1. Operational definitions of framework used in the review. [file 12889_2024_19778_MOESM1_ESM.docx]

**Additional File 1**

Operational definitions of dimensions and abilities of Levesque et al.’s framework on access to health care adapted for this scoping review (1).

| **Health system dimensions** | **Patient abilities** |
| --- | --- |
| ***Approachability***  Existence of services and availability of transparent information on services | ***Ability to perceive***  Understanding of what circumstances require care, linked to health literacy and health beliefs |
| ***Acceptability***  Degree of acceptance of health services among a population within their cultural and social context | ***Ability to seek***  Knowledge about health care options, personal and cultural values related to accessing care, autonomy in seeking care |
| ***Availability and accommodation***  Physical and timely reachability, appropriate number and distribution of services | ***Ability to reach***  Dependent upon patients’ mobility, transport possibilities, and occupational flexibility |
| ***Affordability***  Economic aspect of access to services in terms of direct costs and indirect expenses | ***Ability to pay***  Services can be afforded without catastrophic expenditure through generated income or recourse to savings or borrowings |
| ***Appropriateness***  Fit of services regarding patient’s needs, timeliness, adequate and quality management | ***Ability to engage***  Communication and self-efficacy to be involved in participatory health decision-making |

1. Levesque JF, Harris MF, Russell G. Patient-centred access to health care: conceptualising access at the interface of health systems and populations. International journal for equity in health. 2013;12:18.
