# Supplementary material for: Contraceptive access and use among women with migratory experience living in high-income countries: a scoping review
Source: BMC Public Health. 2024 Sep 20;24:2569. doi: 10.1186/s12889-024-19778-y (PMC11414253; doi:10.1186/s12889-024-19778-y)
Supplement: Supplementary file 2 — Additional File 2. Search strategies for all databases. [file 12889_2024_19778_MOESM2_ESM.docx]

**Additional File 2 – Search strategies for all databases**

1. Medline

| Interface: Ovid MEDLINE(R) and Epub Ahead of Print, In-Process & Other Non-Indexed Citations and Daily  Date of Search: June 30, 2023  Number of hits: 1,370  Comment: In Ovid, two or more words are automatically searched as phrases; i.e. no quotation marks are needed | Field labels   - exp/ = exploded MeSH term - / = non exploded MeSH term - .ti,ab,kf. = title, abstract and author keywords - adjx = within x words, regardless of order - * = truncation of word for alternate endings |
| --- | --- |
| Database(s): **Ovid MEDLINE(R) ALL**1946 to June 29, 2023 Search Strategy:   \| **#** \| **Searches** \| **Results** \| \| --- \| --- \| --- \| \| 1 \| exp Contraception/ \| 29523 \| \| 2 \| exp Reproductive Behavior/ \| 10257 \| \| 3 \| Reproductive Health/ \| 5079 \| \| 4 \| Sexual Health/ \| 2432 \| \| 5 \| exp Contraceptive Agents/ \| 79024 \| \| 6 \| exp Contraceptive Devices/ \| 27060 \| \| 7 \| Reproductive Health Services/ \| 2414 \| \| 8 \| Family Planning Services/ \| 26422 \| \| 9 \| Pregnancy, Unplanned/ \| 2449 \| \| 10 \| Pregnancy, Unwanted/ \| 2710 \| \| 11 \| exp Abortion, Induced/ \| 42764 \| \| 12 \| (abortion* or birth control* or cervical cap* or condom? or contraception* or contraceptive* or family planning or fertility control* or intrauterine device* or reproductive behavio?r* or reproductive health* or sexual health* or vaginal barrier* or vaginal diaphragm* or vaginal ring* or vaginal sponge*).ti,ab,kf. \| 204268 \| \| 13 \| ((plan* or prevent* or unplanned or uninten* or unwant*) adj3 pregnanc*).ti,ab,kf. \| 21454 \| \| 14 \| (fertilization adj2 inhibit*).ti,ab,kf. \| 551 \| \| 15 \| or/1-14 \| 283196 \| \| 16 \| exp Human Migration/ \| 27822 \| \| 17 \| exp "Emigrants and Immigrants"/ \| 15593 \| \| 18 \| "Transients and Migrants"/ \| 14234 \| \| 19 \| Refugees/ \| 13184 \| \| 20 \| Refugee Camps/ \| 295 \| \| 21 \| (alien* or asile or asylum* or (border* adj2 cross*) or (countr* adj3 origin*) or diaspora or displace? or displacement* or emigrant* or emigration or expat? or expatriate? or foreigner* or foreign-born* or foreign background* or foreign population* or immigrant* or immigration or migrant* or migration or naturalized citizen* or new* arriv* or newcomer* or new-comer* or nomad* or non-citizen* or nonnative* or non-native* or nonnational or non-national or nonresident or non-resident* or resettlement* or re-settlement* or refugee* or settler* or squatter* or transient worker* or undocumented worker*).ti,ab,kf. \| 586624 \| \| 22 \| or/16-21 \| 600412 \| \| 23 \| exp Europe/ \| 1547850 \| \| 24 \| exp United States/ \| 1461386 \| \| 25 \| exp Canada/ \| 181629 \| \| 26 \| exp Australia/ \| 170108 \| \| 27 \| New Zealand/ \| 44151 \| \| 28 \| (Andorra* or Australia* or Austria* or Belgian or Baltic or Belgium or British or Canada or Canadian* or Channel Islands or Croatia* or Cypriot or Cyprus or Czech* or Danish or Denmark or England or Estonia* or Europe* or Faroe Islands or Finnish or Finland or France or French or German* or Gibraltar or Greece or Greek* or Greenland* or Hungary or Hungarian or Iceland* or Ireland or Irish or "Isle of Man" or Israel* or Italy or Italian or Latvia* or Liechtenstein or Lithuania* or Luxembourg or Malta or Maltese or Monaco or Netherlands or Dutch or New Zealand* or Nordic Countr* or North America* or Norway or Norwegian* or Poland or Polish or Portugal or Portugues* or Romania* or San Marino or Scandinavia* or Scotland or Scottish or Slovak* or Slovenia* or Spain or Spanish or Sweden or Swedish or Swiss or Switzerland or United Kingdom or United States or Wales or Welsh).ti,ab,kf. \| 2180981 \| \| 29 \| (Alabama or Alaska or Appalachian Region or Arizona or Arkansas or California or Colorado or Connecticut or Delaware or "District of Columbia" or Florida or Georgia or Great Lakes Region or Hawaii or Idaho or Illinois or Indiana or Iowa or Kansas or Kentucky or Louisiana or Maine or Maryland or Massachusetts or Michigan or Mid-Atlantic Region or Minnesota or Mississippi or Missouri or Montana or Nebraska or Nevada or New England or New Hampshire or New Jersey or New Mexico or New York or North Carolina or North Dakota or Ohio or Oklahoma or Oregon or Pacific States or Pennsylvania or Rhode Island or South Carolina or South Dakota or Tennessee or Texas or USA or United States or Utah or Vermont or Virginia or Washington or Wisconsin or Wyoming).ti,ab,kf. \| 847669 \| \| 30 \| (Alberta or British Columbia or Manitoba or New Brunswick or Newfoundland or Northwest Territories or Nova Scotia or Nunavut or Ontario or Prince Edward Island or Quebec or Saskatchewan or Yukon Territory).ti,ab,kf. \| 81868 \| \| 31 \| (New South Wales or Northern Territory or Queensland or Tasmania or Victoria).ti,ab,kf. \| 37903 \| \| 32 \| (high income adj2 (countr* or setting*)).ti,ab,kf. \| 13188 \| \| 33 \| or/23-32 \| 4643541 \| \| 34 \| 15 and 22 and 33 \| 2255 \| \| 35 \| limit 34 to yr="2000 -Current" \| 1481 \| \| 36 \| limit 35 to english language \| 1370 \| | |

2. Web of Science Core Collection

| Interface: Clarivate Analytics  Editions = A&HCI , ESCI , SCI-EXPANDED , SSCI  Date of Search: June 30, 2023  Number of hits: 1,662 | Field labels   - TS/Topic = title, abstract, author keywords and Keywords Plus - NEAR/x = within x words, regardless of order - * = truncation of word for alternate endings   Note: the *Exact search*-function was used for all the searches |
| --- | --- |
| \| **#** \| **Search Query** \| **Results** \| \| --- \| --- \| --- \| \| 1 \| TS=(abortion* OR "birth control*" OR "cervical cap*" OR condom$ OR contraception* OR contraceptive* OR "family planning" OR "fertility control*" OR "intrauterine device*" OR "reproductive behavio$r*" OR "reproductive health*" OR "sexual health*" OR "vaginal barrier*" OR "vaginal diaphragm*" OR "vaginal ring*" OR "vaginal sponge*") \| 201944 \| \| 2 \| TS=((plan* OR prevent* OR unplanned OR uninten* OR unwant* ) NEAR/3 pregnanc*) \| 20256 \| \| 3 \| TS=(fertilization NEAR/2 inhibit*) \| 586 \| \| 4 \| #3 OR #2 OR #1 \| 212300 \| \| 5 \| TS=(alien* OR asile OR asylum* OR (border* NEAR/2 cross* ) OR (countr* NEAR/3 origin* ) OR diaspora OR displace$ OR displacement* OR emigrant* OR emigration OR expat$ OR expatriate$ OR foreigner* OR foreign-born* OR "foreign background*" OR "foreign population*" OR immigrant* OR immigration OR migrant* OR migration OR "naturalized citizen*" OR "new* arriv*" OR newcomer* OR new-comer* OR nomad* OR non-citizen* OR nonnative* OR non-native* OR nonnational OR non-national OR nonresident OR non-resident* OR resettlement* OR re-settlement* OR refugee* OR settler* OR squatter* OR "transient worker*" OR "undocumented worker*") \| 1333881 \| \| 6 \| TS=(Andorra* OR Australia* OR Austria* OR Belgian OR Baltic OR Belgium OR British OR Canada OR Canadian* OR "Channel Islands" OR Croatia* OR Cypriot OR Cyprus OR Czech* OR Danish OR Denmark OR England OR Estonia* OR Europe* OR "Faroe Islands" OR Finnish OR Finland OR France OR French OR German* OR Gibraltar OR Greece OR Greek* OR Greenland* OR Hungary OR Hungarian OR Iceland* OR Ireland OR Irish OR "Isle of Man" OR Israel* OR Italy OR Italian OR Latvia* OR Liechtenstein OR Lithuania* OR Luxembourg OR Malta OR Maltese OR Monaco OR Netherlands OR Dutch OR "New Zealand*" OR "Nordic Countr*" OR "North America*" OR Norway OR Norwegian* OR Poland OR Polish OR Portugal OR Portugues* OR Romania* OR "San Marino" OR Scandinavia* OR Scotland OR Scottish OR Slovak* OR Slovenia* OR Spain OR Spanish OR Sweden OR Swedish OR Swiss OR Switzerland OR "United Kingdom" OR "United States" OR Wales OR Welsh) \| 5935332 \| \| 7 \| TS=(Alabama OR Alaska OR "Appalachian Region" OR Arizona OR Arkansas OR California OR Colorado OR Connecticut OR Delaware OR "District of Columbia" OR Florida OR Georgia OR "Great Lakes Region" OR Hawaii OR Idaho OR Illinois OR Indiana OR Iowa OR Kansas OR Kentucky OR Louisiana OR Maine OR Maryland OR Massachusetts OR Michigan OR "Mid-Atlantic Region" OR Minnesota OR Mississippi OR Missouri OR Montana OR Nebraska OR Nevada OR "New England" OR "New Hampshire" OR "New Jersey" OR "New Mexico" OR "New York" OR "North Carolina" OR "North Dakota" OR Ohio OR Oklahoma OR Oregon OR "Pacific States" OR Pennsylvania OR "Rhode Island" OR "South Carolina" OR "South Dakota" OR Tennessee OR Texas OR USA OR "United States" OR Utah OR Vermont OR Virginia OR Washington OR Wisconsin OR Wyoming) \| 1865502 \| \| 8 \| TS=(Alberta OR "British Columbia" OR Manitoba OR "New Brunswick" OR Newfoundland OR "Northwest Territories" OR "Nova Scotia" OR Nunavut OR Ontario OR "Prince Edward Island" OR Quebec OR Saskatchewan OR "Yukon Territory") \| 203343 \| \| 9 \| TS=("New South Wales" OR "Northern Territory" OR Queensland OR Tasmania OR Victoria) \| 94220 \| \| 10 \| TS=("high income" NEAR/2 (countr* OR setting*)) \| 14641 \| \| 11 \| #6 OR #7 OR #8 OR #9 OR #10 \| 7036215 \| \| 12 \| #4 AND #5 AND #11 \| 1879 \| \| 13 \| #4 AND #5 AND #11 and 2000 or 2001 or 2002 or 2003 or 2004 or 2005 or 2006 or 2007 or 2008 or 2009 or 2010 or 2011 or 2012 or 2013 or 2015 or 2014 or 2016 or 2017 or 2018 or 2019 or 2020 or 2021 or 2022 or 2023 (Publication Years) \| 1749 \| \| 14 \| #4 AND #5 AND #11 and 2000 or 2001 or 2002 or 2003 or 2004 or 2005 or 2006 or 2007 or 2008 or 2009 or 2010 or 2011 or 2012 or 2013 or 2015 or 2014 or 2016 or 2017 or 2018 or 2019 or 2020 or 2021 or 2022 or 2023 (Publication Years) and English (Languages) \| 1662 \| | |

3. Cinahl

| Interface: Ebsco  Date of Search: June 30, 2023  Number of hits: 973 | Field labels   - MH+ = exploded Cinahl Heading - MH = non exploded Cinahl Heading - TI = title - AB = abstract - Nx = within x words, regardless of order - * = truncation of word for alternate endings |
| --- | --- |
| \| **#** \| **Query** \| **Results** \| \| --- \| --- \| --- \| \| S35 \| S14 AND S21 AND S32 \| 973 \| \| S34 \| S14 AND S21 AND S32 \| 984 \| \| S33 \| S14 AND S21 AND S32 \| 1,028 \| \| S32 \| S22 OR S23 OR S24 OR S25 OR S26 OR S27 OR S28 OR S29 OR S30 OR S31 \| 2,061,095 \| \| S31 \| TI ( ("high income" N2 (countr* OR setting*)) ) OR AB ( ("high income" N2 (countr* OR setting*)) ) \| 5,512 \| \| S30 \| TI ( ("New South Wales" OR "Northern Territory" OR Queensland OR Tasmania OR Victoria) ) OR AB ( ("New South Wales" OR "Northern Territory" OR Queensland OR Tasmania OR Victoria) ) \| 21,835 \| \| S29 \| TI ( (Alberta OR "British Columbia" OR Manitoba OR "New Brunswick" OR Newfoundland OR "Northwest Territories" OR "Nova Scotia" OR Nunavut OR Ontario OR "Prince Edward Island" OR Quebec OR Saskatchewan OR "Yukon Territory") ) OR AB ( (Alberta OR "British Columbia" OR Manitoba OR "New Brunswick" OR Newfoundland OR "Northwest Territories" OR "Nova Scotia" OR Nunavut OR Ontario OR "Prince Edward Island" OR Quebec OR Saskatchewan OR "Yukon Territory") ) \| 38,352 \| \| S28 \| TI ( (Alabama OR Alaska OR "Appalachian Region" OR Arizona OR Arkansas OR California OR Colorado OR Connecticut OR Delaware OR "District of Columbia" OR Florida OR Georgia OR "Great Lakes Region" OR Hawaii OR Idaho OR Illinois OR Indiana OR Iowa OR Kansas OR Kentucky OR Louisiana OR Maine OR Maryland OR Massachusetts OR Michigan OR "Mid-Atlantic Region" OR Minnesota OR Mississippi OR Missouri OR Montana OR Nebraska OR Nevada OR "New England" OR "New Hampshire" OR "New Jersey" OR "New Mexico" OR "New York" OR "North Carolina" OR "North Dakota" OR Ohio OR Oklahoma OR Oregon OR "Pacific States" OR Pennsylvania OR "Rhode Island" OR "South Carolina" OR "South Dakota" OR Tennessee OR Texas OR USA OR "United States" OR Utah OR Vermont OR Virginia OR Washington OR Wisconsin OR Wyoming) ) OR AB ( (Alabama OR Alaska OR "Appalachian Region" OR Arizona OR Arkansas OR California OR Colorado OR Connecticut OR Delaware OR "District of Columbia" OR Florida OR Georgia OR "Great Lakes Region" OR Hawaii OR Idaho OR Illinois OR Indiana OR Iowa OR Kansas OR Kentucky OR Louisiana OR Maine OR Maryland OR Massachusetts OR Michigan OR "Mid-Atlantic Region" OR Minnesota OR Mississippi OR Missouri OR Montana OR Nebraska OR Nevada OR "New England" OR "New Hampshire" OR "New Jersey" OR "New Mexico" OR "New York" OR "North Carolina" OR "North Dakota" OR Ohio OR Oklahoma OR Oregon OR "Pacific States" OR Pennsylvania OR "Rhode Island" OR "South Carolina" OR "South Dakota" OR Tennessee OR Texas OR USA OR "United States" OR Utah OR Vermont OR Virginia OR Washington OR Wisconsin OR Wyoming) ) \| 447,633 \| \| S27 \| TI ( (Andorra* OR Australia* OR Austria* OR Belgian OR Baltic OR Belgium OR British OR Canada OR Canadian* OR "Channel Islands" OR Croatia* OR Cypriot OR Cyprus OR Czech* OR Danish OR Denmark OR England OR Estonia* OR Europe* OR "Faroe Islands" OR Finnish OR Finland OR France OR French OR German* OR Gibraltar OR Greece OR Greek* OR Greenland* OR Hungary OR Hungarian OR Iceland* OR Ireland OR Irish OR "Isle of Man" OR Israel* OR Italy OR Italian OR Latvia* OR Liechtenstein OR Lithuania* OR Luxembourg OR Malta OR Maltese OR Monaco OR Netherlands OR Dutch OR "New Zealand*" OR "Nordic Countr*" OR "North America*" OR Norway OR Norwegian* OR Poland OR Polish OR Portugal OR Portugues* OR Romania* OR "San Marino" OR Scandinavia* OR Scotland OR Scottish OR Slovak* OR Slovenia* OR Spain OR Spanish OR Sweden OR Swedish OR Swiss OR Switzerland OR "United Kingdom" OR "United States" OR Wales OR Welsh) ) OR AB ( (Andorra* OR Australia* OR Austria* OR Belgian OR Baltic OR Belgium OR British OR Canada OR Canadian* OR "Channel Islands" OR Croatia* OR Cypriot OR Cyprus OR Czech* OR Danish OR Denmark OR England OR Estonia* OR Europe* OR "Faroe Islands" OR Finnish OR Finland OR France OR French OR German* OR Gibraltar OR Greece OR Greek* OR Greenland* OR Hungary OR Hungarian OR Iceland* OR Ireland OR Irish OR "Isle of Man" OR Israel* OR Italy OR Italian OR Latvia* OR Liechtenstein OR Lithuania* OR Luxembourg OR Malta OR Maltese OR Monaco OR Netherlands OR Dutch OR "New Zealand*" OR "Nordic Countr*" OR "North America*" OR Norway OR Norwegian* OR Poland OR Polish OR Portugal OR Portugues* OR Romania* OR "San Marino" OR Scandinavia* OR Scotland OR Scottish OR Slovak* OR Slovenia* OR Spain OR Spanish OR Sweden OR Swedish OR Swiss OR Switzerland OR "United Kingdom" OR "United States" OR Wales OR Welsh) ) \| 739,517 \| \| S26 \| (MH "New Zealand") \| 31,591 \| \| S25 \| (MH "Australia+") \| 127,600 \| \| S24 \| (MH "Canada+") \| 111,235 \| \| S23 \| (MH "United States+") \| 746,629 \| \| S22 \| (MH "Europe+") \| 649,727 \| \| S21 \| S15 OR S16 OR S17 OR S18 OR S19 OR S20 \| 100,052 \| \| S20 \| TI ( (alien* OR asile OR asylum* OR (border* N2 cross*) OR (countr* N3 origin*) OR diaspora OR displace# OR displacement* OR emigrant* OR emigration OR expat# OR expatriate# OR foreigner* OR foreign-born* OR "foreign background*" OR "foreign population*" OR immigrant* OR immigration OR migrant* OR migration OR "naturalized citizen*" OR "new* arriv*" OR newcomer* OR new-comer* OR nomad* OR non-citizen* OR nonnative* OR non-native* OR nonnational OR non-national OR nonresident OR non-resident* OR resettlement* OR re-settlement* OR refugee* OR settler* OR squatter* OR "transient worker*" OR "undocumented worker*") ) OR AB ( (alien* OR asile OR asylum* OR (border* N2 cross*) OR (countr* N3 origin*) OR diaspora OR displace# OR displacement* OR emigrant* OR emigration OR expat# OR expatriate# OR foreigner* OR foreign-born* OR "foreign background*" OR "foreign population*" OR immigrant* OR immigration OR migrant* OR migration OR "naturalized citizen*" OR "new* arriv*" OR newcomer* OR new-comer* OR nomad* OR non-citizen* OR nonnative* OR non-native* OR nonnational OR non-national OR nonresident OR non-resident* OR resettlement* OR re-settlement* OR refugee* OR settler* OR squatter* OR "transient worker*" OR "undocumented worker*") ) \| 90,863 \| \| S19 \| (MH "Refugee Camps") \| 214 \| \| S18 \| (MH "Refugees") \| 9,075 \| \| S17 \| (MH "Transients and Migrants") \| 5,810 \| \| S16 \| (MH "Emigration and Immigration") \| 7,394 \| \| S15 \| (MH "Immigrants+") \| 18,053 \| \| S14 \| S1 OR S2 OR S3 OR S4 OR S5 OR S6 OR S7 OR S8 OR S9 OR S10 OR S11 OR S12 OR S13 \| 102,256 \| \| S13 \| TI (fertilization N2 inhibit*) OR AB (fertilization N2 inhibit*) \| 6 \| \| S12 \| TI ( ((plan* OR prevent* OR unplanned OR uninten* OR unwant*) N3 pregnanc*) ) OR AB ( ((plan* OR prevent* OR unplanned OR uninten* OR unwant*) N3 pregnanc*) ) \| 10,404 \| \| S11 \| TI ( (abortion* OR "birth control*" OR "cervical cap*" OR condom# OR contraception* OR contraceptive* OR "family planning" OR "fertility control*" OR "intrauterine device*" OR "reproductive behavio#r*" OR "reproductive health*" OR "sexual health*" OR "vaginal barrier*" OR "vaginal diaphragm*" OR "vaginal ring*" OR "vaginal sponge*") ) OR AB ( (abortion* OR "birth control*" OR "cervical cap*" OR condom# OR contraception* OR contraceptive* OR "family planning" OR "fertility control*" OR "intrauterine device*" OR "reproductive behavio#r*" OR "reproductive health*" OR "sexual health*" OR "vaginal barrier*" OR "vaginal diaphragm*" OR "vaginal ring*" OR "vaginal sponge*") ) \| 62,588 \| \| S10 \| (MH "Abortion, Induced+") \| 11,735 \| \| S9 \| (MH "Pregnancy, Unwanted") \| 1,209 \| \| S8 \| (MH "Pregnancy, Unplanned") \| 2,695 \| \| S7 \| (MH "Family Planning+") \| 13,007 \| \| S6 \| (MH "Contraceptive Devices+") \| 13,084 \| \| S5 \| (MH "Contraceptive Agents+") \| 24,978 \| \| S4 \| (MH "Sexual Health") \| 8,565 \| \| S3 \| (MH "Reproductive Health") \| 9,653 \| \| S2 \| (MH "Reproductive Behavior") \| 51 \| \| S1 \| (MH "Contraception+") \| 12,061 \| | |
