# Supplementary material for: Contraceptive access and use among women with migratory experience living in high-income countries: a scoping review
Source: BMC Public Health. 2024 Sep 20;24:2569. doi: 10.1186/s12889-024-19778-y (PMC11414253; doi:10.1186/s12889-024-19778-y)
Supplement: Supplementary file 3 — Additional File 3. Overview of included articles. [file 12889_2024_19778_MOESM3_ESM.docx]

**Additional File 3**

Overview of included articles (in order of appearance in the review). When the study population consisted of several subgroups (e.g. first- and second-generation), and subgroups had been stratified for, only the subgroup relevant to this review is listed.

| First author, year of publication | Country | Study design and data collection method | Study population (n) | Study aim/objective |
| --- | --- | --- | --- | --- |
| Agbemenu, 2018 | USA | Qualitative; demographic survey, focus groups | Somali Bantu refugee women, aged 18+ (n) | To explore Somali Bantu refugee women’s reproductive health decision-making, as influenced by their resettlement in the USA. |
| Agbemenu, 2020 | USA | Quantitative, cross-sectional; convenience survey | Community-dwelling African refugee women, aged 18-50 (100) | To understand beliefs and attitudes towards family planning and related behaviours, which can impact perinatal health. |
| Akerman, 2016 | Sweden | Quantitative, cross-sectional; postal questionnaires | Thai immigrant women, aged 18-64 (804) | To investigate the association between knowledge and utilization of sexual and reproductive healthcare services, contraceptive knowledge and socio-demographic characteristics and social capital among Thai immigrant women in Sweden. |
| Akerman, 2019 | Sweden | Quantitative, cross-sectional; self-reported questionnaires | Immigrant women born outside the EU, aged 18+ (288) | To investigate the knowledge about and use of sexual and reproductive healthcare services among immigrant women in Sweden. |
| Akerman, 2021 | Sweden | Quantitative, cross-sectional; postal questionnaires | Thai-born women residing in Sweden, aged 23-60 (266) | To investigate the need and use of SRH-related information and services among Thai women residing in Sweden. |
| Alvarez-Nieto, 2015 | Spain | Qualitative; semi-structured, in-depth interviews | Immigrant women residing in Andalusia, aged 16-46 (13) | To shed light on the sexual and reproductive health beliefs and experiences of female immigrants in a region of southern Spain. |
| Barona-Vilar, 2013 | Spain | Qualitative; focus group discussions | First-generation migrant mothers from Bolivia or Ecuador, aged 20-35 (26); midwives (24) | To explore the experiences and perceptions of parenthood and maternal health care among Latin American women living in Spain. |
| Betancourt, 2013 | USA | Mixed Methods; focus group discussions, structured individual interviews | Mexico-born women living in New York (151) | To document SRH care utilization and barriers to accessing care for female Mexican immigrants living in NYC, and investigate the association between knowledge, barriers, years in the US, and having health insurance and accessing health care. |
| Burchard, 2011 | Australia | Qualitative; focus group discussions | Female students from China and Malaysia (21) | To gain a deeper understanding of the sexual health knowledge and practices of female international students at the University of Adelaide in South Australia. |
| Caballero, 2022 | USA | Quantitative, cross-sectional; orally administered survey | Spanish-speaking Latina immigrant mothers, aged 18-50 (194) | To characterise contraceptive method use and satisfaction among Spanish-speaking Latina immigrants who attend their child's well care visit. |
| Carvajal, 2017 | USA | Qualitative; focus group discussions, individual semi-structured interviews | Latina immigrant women, aged 15-24 (16) | To describe Latinas' perspectives regarding specific factors that influence their contraceptive decision making and to describe their perspectives about the role of primary care physicians in the decision making. |
| Carvajal, 2020 | USA | Qualitative; focus group discussion, semi-structured interviews | Foreign-born Latina women, aged 15-24 (16) | To identify how the lived experiences of young Latinas impact family planning and childbearing decisions within the context of their life plans. |
| Choi, 2016 | USA | Quantitative, cross-sectional; National Survey on Family Growth (NSFG) | Mexican immigrant women, aged 15-44 (229) | To describe racial, ethnic, and nativity disparities in contraceptive practices and determine the relative importance of the various mechanisms proposed to explain those disparities among never married, non-cohabiting women. |
| Coleman-Minahan, 2019 | USA | Quantitative, prospective cohort study; interview survey | Hispanic foreign-born women, aged 18-44 (502) | To examine the association between quality of postpartum contraceptive counselling and changes in contraceptive method preference between delivery and 3-months postpartum. |
| Coleman-Minahan, 2022 | USA | Quantitative, cross-sectional; national survey (NSFG) | Foreign-born female survey respondents of Mexican origin in the US, aged 15-44 (719) | To examine current contraceptive use by parity among four ethnicity and nativity groups. |
| Craig, 2014 | USA | Quantitative, cross-sectional; survey | Foreign-born female respondents, aged 18-29 (75) | To explore racial/ethnic disparities in specific areas of contraceptive knowledge. |
| Degni, 2006 | Finland | Mixed Methods; questionnaires, interviews | Somali refugee women, aged 18-50 (70) | To assess attitudes towards and perceptions about contraceptive use among married refugee women of Somali descent living in Finland. |
| Dolan, 2020 | Australia | Qualitative; semi-structured in-depth interviews | Healthcare providers (20) | To explore healthcare providers' experiences of providing contraceptive care for Chinese migrant women, their perceptions of women's care needs when choosing contraceptive methods, as well as their own needs in supporting women's decision-making. |
| Dolan, 2021 | Australia | Qualitative; semi-structured interviews | Chinese women living in Australia, aged 18-45 (22) | To explore Chinese women's knowledge, perceptions and views of contraceptive methods, and their experiences with choosing one in Australia using a qualitative critical realist approach. |
| Dolan, 2022 | Australia | Qualitative; semi-structured interviews | Chinese migrant women, aged 18-45 (22); healthcare professionals (20) | To explore the perceived acceptability, usefulness, and feasibility of a suite of encounter decision aids (DAs) on contraceptive methods with Chinese migrant women living in Australia and healthcare providers. |
| Ebrahim, 2017 | USA | Quantitative, cross-sectional; questionnaires | Somali and Ethiopian immigrant women (90) | To examine the main predictors of male condom use in steady heterosexual relationships among Somali and Ethiopian immigrants in the U.S. |
| Emtell Iwarsson, 2019 | Sweden | Quantitative, cross-sectional; questionnaires | First-generation migrant women, aged 18+ (148) | To compare ever-in life contraception use, use of contraception at current conception, and planned use of contraception after induced abortion and to compare types of contraception methods used and intended for future use. |
| Emtell Iwarsson, 2022 | Sweden | Quantitative, cluster randomised controlled trial; questionnaires | Foreign-born migrant women, aged 18+ (169) | To evaluate effects of structured contraceptive counselling among non-migrants, foreign-born migrants and second-generation migrants. |
| Flippen, 2022 | USA | Quantitative, cross-sectional; surveys | Mexican-born women who had migrated to Durham, aged 18-49 (526) | To assess how selection, disruption and adaptation shape contraceptive use among Mexican migrant women. |
| Garcés-Palacio, 2008 | USA | Quantitative, cross-sectional; questionnaires | Immigrant Hispanic women, aged 18-42 (113) | To examine the differences between low-income Hispanic immigrants' and low-income non-Hispanics' contraceptive use, reproduction and contraception knowledge, demographic and knowledge factors associated with their choice of contraceptive. |
| Gele, 2019 | Norway | Quantitative, cross-sectional; questionnaires | Somali immigrant women in Oslo (228) | To investigate the unmet need for contraception among Somali immigrant women in Oslo, Norway, compared to their original population in Mogadishu, Somalia. |
| Gele, 2020 | Norway | Qualitative; unstructured, in-depth interviews | First-generation Somali immigrant women, aged 18+ (21) | To explore the barriers and facilitators to contraceptive usage among Somali immigrant women in Oslo area. |
| Goldenberg, 2023 | Canada | Qualitative; in-depth interviews | Im/migrant women, aged 18-49 (78); im/migrant-focused service providers (10) | To describe lived experiences of health insurance coverage and the health and social impacts of gaps in health insurance amongst im/migrant women in British Columbia (BC), Canada. |
| Grassby, 2021 | Canada | Qualitative; in-depth interviews | Im/migrant women, aged 15-49 (47); service providers (10) | To evaluate impacts of a residency-based waiting period for health insurance coverage on lived experiences of health and settlement for im/migrant women in British Columbia, Canada. |
| Guerra-Reyes, 2021 | USA | Qualitative; semi-structured, in-depth interviews | Latina-identified women, aged 25-65 (15) | To understand the complexities of sexual and reproductive health care access for Latinas in an emerging Latino community. |
| Gurnah, 2011 | USA | Qualitative; interviews, focus group discussions, semi-structured surveys | Somali Bantu women, aged 22-45 (10); service providers (5) | To explore the reproductive health experiences of Somali Bantu women in Connecticut and to identify potential barriers to care experienced by marginalised populations. |
| Hawkey, 2018 | Australia | Qualitative; individual interviews, focus group discussions | Migrant and refugee women in Australia or Canada, aged 18+ (160) | To explore experiences and constructions of fertility and fertility control among new migrant and refugee women in Sydney, Australia and Vancouver, Canada. |
| Inci, 2020 | Germany | Quantitative, cross-sectional; semi-structured questionnaires | Refugee women, aged 18+ (307) | To describe the current reproductive health status of female refugees and to provide an initial overview of their existing unmet family planning and contraception needs. |
| Inoue, 2016 | Australia | Qualitative; in-depth interviews | Japanese migrant women, aged mid-20ies to mid-40ies (7) | To report on findings from a cohort of migrant Japanese women who participated in a study of Australian women's understanding and experience of contraceptives. |
| Kilander, 2022 | Sweden | Mixed Methods, organisational case study; meetings notes, emails, telephone calls, seminar notes, questionnaires, registry data | Midwives at three maternal health clinics in Stockholm (n unknown); user feedback from one recently pregnant immigrant woman and one immigrant couple | To evaluate a Quality Improvement Collaborative (QIC) whose objective was to improve contraceptive services for immigrant women postpartum, through health care professionals' counselling and resulting in women choosing a more effective contraceptive method. |
| Kolak, 2017 | Sweden | Qualitative; semi-structured interviews | Midwives to immigrant women (10) | To describe midwives' experiences of providing contraception counselling to immigrant women. |
| Kolak, 2022 | Sweden | Qualitative; in-depth semi-structured interviews | Foreign-born immigrant women resident in Sweden, aged 18-49 (19) | To explore immigrant women's perspectives on contraceptive counselling provided by midwives in Sweden. |
| Larsson, 2016 | Sweden | Qualitative; individual interviews | Midwives (10) and female medical doctors (3) working at abortion clinics | To explore health care providers' experiences of providing care to immigrant women seeking abortion care. |
| Lauria, 2014 | Italy | Quantitative, cross-sectional; population-based surveys | Immigrant resident women (583) | To describe the use of contraception in a sample of Italian and immigrant women three months after they had delivered a baby, to identify factors associated with its use and, in particular, to evaluate the effect of having received information on contraception during pregnancy and postpartum care. |
| Lee, 2011 | USA | Quantitative, cross-sectional; telephone survey | Korean immigrant women, aged 18-55 (145) | To explore within a theoretical context the relationship between acculturation and oral contraceptive use among Korean immigrant women. |
| Maternowska, 2010 | USA | Qualitative; semi-structured interviews | Mexican-born women, aged 18-39 (26) | To present findings from qualitative work related to the experiences growing body of migration literature that see culture, health and sexuality change on a continuum. |
| Marchetti, 2023 | Italy | Qualitative; focus group discussions | Asylum seeker and refugee women (25) | To explore the health needs in their bio-psycho-social meaning, and the quality of health care as perceived from the ASRs’ perspective. |
| Newbold, 2009 | Canada | Qualitative; key informant interviews | Female healthcare professionals (9) | To understand issues that healthcare professionals face in the delivery of family planning and reproductive health services among immigrants, with results revealing the complexity of providing care to this population. |
| Ngum Chi Watts, 2014 | Australia | Qualitative; in-depth interviews | African Australian women, aged 17-30 (16) | To discuss the contraceptive knowledge and attitudes of teenagers and women of African descent who have experienced teenage pregnancy, including the role of myths and misinformation in regard to contraceptive use. |
| Olorunsaiye, 2023 | USA | Quantitative, cross-sectional; online survey | Foreign-born Black women, aged 18-44 (98) | To assess the contraceptive attitudes of Black US-born and foreign-born women living in the USA and to examine differences by nativity. |
| Omland, 2014 | Norway | Quantitative, cross-sectional; registry-based | Foreign-born immigrant women, aged 16-45 (130 080) | To examine the use of hormonal contraceptives among immigrant and native women in Norway. |
| Otero-Garcia, 2013 | Spain | Qualitative; in-depth interviews | Midwives in primary care (7) | To explore the perceptions of midwives who provide these services regarding immigrant women’s access and participation in sexual and reproductive health programs offered in a rural area. |
| Poncet, 2013 | France | Quantitative, cross-sectional; questionnaires |  | To investigate the extent to which educational attainment, type of occupation and household income are related to the use of contraception and choice of contraceptive method. |
| Quelopana, 2014 | USA | Qualitative; focus group discussions | Immigrant Hispanic women, aged 18+ (24) | To gain a better understanding of the process of transformation of immigrant women’s knowledge, beliefs and experiences around sexual and reproductive health as a result of living in the US. |
| Raben, 2018 | Netherlands | Quantitative, retrospective, cross-sectional study; electronic medical records | First-generation migrant and refugee women born outside the EU, aged 15-49 (162) | To get insight into general practitioner care related to contraception in refugees and migrants compared with native Dutch women. |
| Rasch, 2007 | Denmark | Quantitative, case control study; questionnaires | Immigrant women, aged 15-39 (242) | To elucidate how contraceptive knowledge and attitudes among Danish-born and immigrant women influence them in their choice of abortion. |
| Rodriguez, 2021 | USA | Quantitative, retrospective cohort study; Medicaid claims and birth certificate data | Women covered by Emergency Medicaid, aged 15-44 (23 971) | To examine the association of a policy extending postpartum coverage to Emergency Medicaid recipients with attendance at postpartum visits and use of postpartum contraception. |
| Rodriguez, 2022 | USA | Quantitative, retrospective cohort study; Medicaid claims and birth certificate data | Women covered by Emergency Medicaid, aged 15-44 (42 391) | To understand how differences in the type of Medicaid (Traditional vs Emergency) are associated with postpartum care attendance and contraceptive use. |
| Royer, 2020 | USA | Qualitative; focus group discussions | Somali and Congolese resettled refugee women, aged 18-68 (66) | To address gaps in the literature regarding refugee women’s family planning knowledge, attitudes, and practices after third country resettlement to the U.S. |
| Russo, 2020 | Australia | Qualitative; focus group discussions, semi-structured interviews | Afghan refugee women living in Melbourne, aged 18-49 (28) | To explore the family planning perspectives and experiences of Afghan women and men living in Melbourne, Australia. |
| Sable, 2009 | USA | Qualitative; focus group discussions | Hispanic foreign-born women residing in the US, aged 18-51 (32) | To identify factors that present barriers to accessing and using family planning services and contraceptives among recent female Hispanic immigrants to a small Midwestern community. |
| Sangi-Haghpeykar, 2006 | USA | Quantitative, cross-sectional; self-administered questionnaires | Non-US-born Hispanic low-income women (231) | Not clearly stated. |
| Sargent, 2005 | France | Mixed Methods; interviews, observations, surveys | Midwives serving Mali women (n unknown) | To explore how the personal, state, and global articulate in shaping reproductive strategies and relations in the Malian migrant population. |
| Soin, 2020 | USA | Qualitative; interviews, focus group discussions | Refugee women from Bhutan/Nepal, Burma, Iraq, aged 18+ (32) | To learn about the family planning practices of resettled refugees and the factors that promote and/or hinder contraceptive use. |
| Sudhinaraset, 2023 | USA | Qualitative; in-depth, semi-structured interviews | Chinese and Mexican immigrant women, aged 26-46 (18) | To examine how language access may influence immigrant women’s experiences and quality of care. |
| Ussher, 2012 | Australia | Qualitative; focus group discussions | Women members of the Assyrian and Karen communities in Australia with refugee background, aged 18-78 (42) | To examine constructions and experiences of reproductive and sexual health and associated services in two cultural groups whose experiences have not previously been examined: Assyrian and Karen women from Western Sydney, who arrived in Australia as refugees. |
| Verran, 2015 | UK | Qualitative; semi-structured interviews | Female Chinese asylum seekers, aged 26-41 (10) | To explore Chinese asylum seekers' knowledge and understanding of family planning once living in the UK. |
| Watts, 2015 | Australia | Qualitative, focus group discussions, in-depth interviews | African Australian migrant mothers, aged 17-30 (16); service providers (5) | To examine contraception awareness and use among African Australian women in Melbourne, Australia, who have experienced teenage pregnancy, and to explore the social contexts that shape these women’s attitudes towards contraception. |
| White, 2017 | USA | Qualitative; in-depth interviews | Latina immigrant women, aged 19-44 (20) | To examine the interrelationships of the multiple factors related to women’s contraceptive use, using the Social Ecological Model (SEM). |
| Wiebe, 2013 | Canada | Quantitative, cross-sectional; questionnaires | Immigrant women (533) | To compare experiences, attitudes, and beliefs of immigrant and non-immigrant women presenting for abortion with regard to contraception, and to identify difficulties involved in accessing contraception in Canada. |
| Wolff, 2008 | Switzerland | Quantitative, prospective cohort study; questionnaires | Undocumented pregnant women (161) | To describe detailed information about contraception, intendedness of pregnancy, health status and behaviour, violence exposure, and birth outcomes of undocumented migrant women and to compare them to those of women having a legal residence permit. |
| Wray, 2014 | Australia | Qualitative; in-depth, semi-structured interviews | Muslim immigrant women, aged 18-25 (10) | To investigate young Muslim women migrants’ accounts of sexual health. |
| Zhang, 2020 | USA | Qualitative; focus group discussions | Somali refugee women living in the US, aged 18-49 (53) | To explore Somali women’s knowledge, attitudes, and experiences with birth spacing and contraception. |
