# Supplementary material for: Contraceptive access and use among women with migratory experience living in high-income countries: a scoping review
Source: BMC Public Health. 2024 Sep 20;24:2569. doi: 10.1186/s12889-024-19778-y (PMC11414253; doi:10.1186/s12889-024-19778-y)
Supplement: Supplementary file 4 — Additional File 4. Brief description of identified interventions related to contraceptive counselling. [file 12889_2024_19778_MOESM4_ESM.docx]

**Additional File 4**

| Author, country, year | Description of intervention | Description of study | Dimensions of Levesque et al.’s framework |
| --- | --- | --- | --- |
| Dolan et al., Australia, 2022 | Interview study with Chinese migrant women living in Australia and HCPs as part of a larger trial on interventions for facilitating shared decision-making in contraceptive counselling – exploration of perceptions of birth control patient Decision Aids (DAs). | DAs were found to be helpful in delivering information on different methods and in the Chinese language; improvements were suggested in terms of adding information on cost to the DAs and disseminating them also outside of clinics and through social media channels. | “Approachability”  “Acceptability”  “Affordability”  “Appropriateness” |
| Emtell Iwarsson et al., Sweden, 2022 | Cluster randomised controlled trial focusing on structured contraceptive counselling for women seeking contraceptive care (foreign-born women as one subpopulation) through an educational video, key questions, and educational tools. | High satisfaction among foreign-born women. | “Appropriateness” |
| Kilander et al., Sweden, 2022 | Pilot study of a Quality Improvement Collaborative (QIC) involving midwives and immigrant women; implementation of changes in scheduling practices (double contraceptive counselling sessions, routine bookings of postpartum visits during pregnancy) and in communication techniques. | Choice of more effective contraception increased in the group of immigrant women. Identification of need for better counselling tools for women not proficient in Swedish on behalf of the midwives. | “Availability and accommodation”  “Appropriateness” |

Brief description of included articles on interventions related to contraception.
